# Amidochelocardin Overcomes Resistance Mechanisms Exerted on Tetracyclines and Natural Chelocardin

**DOI:** 10.3390/antibiotics9090619

**Published:** 2020-09-18

**Authors:** Fabienne Hennessen, Marcus Miethke, Nestor Zaburannyi, Maria Loose, Tadeja Lukežič, Steffen Bernecker, Stephan Hüttel, Rolf Jansen, Judith Schmiedel, Moritz Fritzenwanker, Can Imirzalioglu, Jörg Vogel, Alexander J. Westermann, Thomas Hesterkamp, Marc Stadler, Florian Wagenlehner, Hrvoje Petković, Jennifer Herrmann, Rolf Müller

**Affiliations:** 1 Department of Microbial Natural Products, Helmholtz Institute for Pharmaceutical Research Saarland (HIPS)—Helmholtz Centre for Infection Research (HZI), and Department of Pharmacy, Saarland University Campus E8.1, 66123 Saarbrücken, Germany; fabienne.hennessen@nano-care.de (F.H.); Marcus.Miethke@helmholtz-hips.de (M.M.); nestor.zaburannyi@helmholtz-hips.de (N.Z.); Tadeja.Lukezic@nib.si (T.L.); 2 German Center for Infection Research (DZIF), Partner Site Hannover-Braunschweig, 38124 Braunschweig, Germany; Steffen.Bernecker@helmholtz-hzi.de (S.B.); Stephan.Huettel@helmholtz-hzi.de (S.H.); Rolf.Jansen@helmholtz-hzi.de (R.J.); Thomas.Hesterkamp@helmholtz-hzi.de (T.H.); Marc.Stadler@helmholtz-hzi.de (M.S.); 3 Clinic for Urology, Paediatric Urology & Andrology, Justus-Liebig University Gießen, and German Center for Infection Research (DZIF), Partner Site Giessen-Marburg-Langen, 35392 Gießen, Germany; Maria.Loose@chiru.med.uni-giessen.de (M.L.); Florian.Wagenlehner@chiru.med.uni-giessen.de (F.W.); 4 National Institute of Biology, Večna pot 111, 1000 Ljubljana, Slovenia; 5 Department of Microbial Drugs, Helmholtz Centre for Infection Research (HZI), Inhoffenstrasse 7, 38124 Braunschweig, Germany; 6 Institute of Medical Microbiology, Justus-Liebig University Gießen, and German Center for Infection Research (DZIF), Partner Site Giessen-Marburg-Langen, 35390 Gießen, Germany; Judith.Schmiedel@mikrobio.med.uni-giessen.de (J.S.); Moritz.Fritzenwanker@mikrobio.med.uni-giessen.de (M.F.); Can.Imirzalioglu@mikrobio.med.uni-giessen.de (C.I.); 7 Helmholtz Institute for RNA-based Infection Research (HIRI), Helmholtz Centre for Infection Research (HZI) and Institute of Molecular Infection Biology (IMIB), University of Würzburg, Josef-Schneider-Str. 2, 97080 Würzburg, Germany; joerg.vogel@helmholtz-hiri.de (J.V.); alexander.westermann@helmholtz-hiri.de (A.J.W.); 8 Department of Food Science and Technology, Biotechnical Faculty, University of Ljubljana, Jamnikarjeva 101, 1000 Ljubljana, Slovenia; Hrvoje.Petkovic@bf.uni-lj.si

**Keywords:** chelocardins, atypical tetracyclines, broad-spectrum antibiotics, clinical isolates, uropathogens, urinary tract infection (UTI), resistance-breaking properties, mechanism of resistance, AcrAB-TolC efflux pump

## Abstract

The reassessment of known but neglected natural compounds is a vital strategy for providing novel lead structures urgently needed to overcome antimicrobial resistance. Scaffolds with resistance-breaking properties represent the most promising candidates for a successful translation into future therapeutics. Our study focuses on chelocardin, a member of the atypical tetracyclines, and its bioengineered derivative amidochelocardin, both showing broad-spectrum antibacterial activity within the ESKAPE (*Enterococcus faecium, Staphylococcus aureus, Klebsiella pneumoniae, Acinetobacter baumannii, Pseudomonas aeruginosa*, and *Enterobacter* species) panel. Further lead development of chelocardins requires extensive biological and chemical profiling to achieve favorable pharmaceutical properties and efficacy. This study shows that both molecules possess resistance-breaking properties enabling the escape from most common tetracycline resistance mechanisms. Further, we show that these compounds are potent candidates for treatment of urinary tract infections due to their in vitro activity against a large panel of multidrug-resistant uropathogenic clinical isolates. In addition, the mechanism of resistance to natural chelocardin was identified as relying on efflux processes, both in the chelocardin producer *Amycolatopsis sulphurea* and in the pathogen *Klebsiella pneumoniae.* Resistance development in *Klebsiella* led primarily to mutations in *ramR*, causing increased expression of the *acrAB-tolC* efflux pump. Most importantly, amidochelocardin overcomes this resistance mechanism, revealing not only the improved activity profile but also superior resistance-breaking properties of this novel antibacterial compound.

## 1. Introduction

The ongoing emergence of antimicrobial resistance (AMR) and the concurrent decline in effective treatment options is a severe threat for the human population. If no solutions are provided to arrest the drop in developing new effective drugs, the number of deaths due to AMR was predicted to increase up to 10 million per year in 2050 [1]. This problem is accompanied by the rising evolution of multidrug-resistant (MDR) bacteria, which are described especially among hard-to-treat pathogens belonging to the ESKAPE group (*Enterococcus faecium*, *Staphylococcus aureus*, *Klebsiella pneumoniae*, *Acinetobacter baumannii*, *Pseudomonas aeruginosa*, and *Enterobacter* species) [2,3,4]. Gram-negative bacteria such as *Enterobacteriaceae*, *A. baumannii*, and *P. aeruginosa* have been emphasized by the World Health Organization (WHO) as the most critical pathogens for which new treatments need to be prioritized [5].

For past decades, the main source for the development of new pharmaceuticals in general, especially antibiotics, are natural products (NPs) mainly derived from plants, fungi, and bacteria [6]. Among the latter, actinobacteria, in particular *Streptomyces* spp., represent the most relevant producers of known antibacterial NPs [7,8]. However, the number of new antibacterial compounds isolated from these bacterial producers drops continuously, hampering the future development of novel drug candidates [9,10]. To overcome this shortage, alternative strategies in drug development are applied. The reassessment of already known and effective, but yet neglected NP scaffolds has become a promising route to help fuel the dwindling antibiotic development pipeline [11,12,13]. Chemical and biotechnological approaches such as semisynthesis or genetic engineering, respectively, are applied to modify such molecules, aiming to improve their pharmaceutical properties, antibacterial spectra, and efficacies [14].

**Figure 1:**
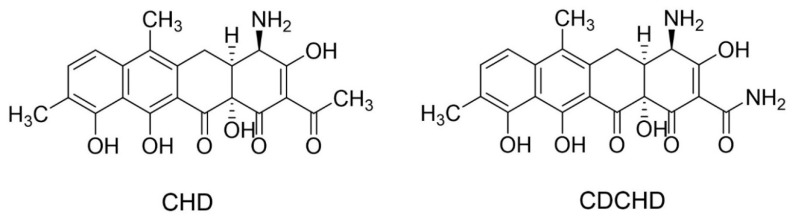
Chemical structures of chelocardin (CHD) [28] and its amidated derivative 2-carboxamido-2-deacetyl-chelocardin (amidochelocardin, CDCHD) [15].

Amidochelocardin (2-carboxamido-2-deacetyl-chelocardin, CDCHD; Figure 1) is a promising example of a new lead structure gained by biosynthetic engineering of the bioactive NP chelocardin (CHD; Figure 1), also known as M-319, cetocycline, or cetotetrine [15]. CHD is produced by the actinomycete *Amycolatopsis sulphurea* and was described in the early 1960s [16]. As a member of the atypical tetracyclines, it exhibits a broad-spectrum antibacterial activity, including the inhibition of species causing urinary tract infections (UTIs), while showing significant differences to the common mode of action of tetracyclines [17,18,19,20]. Importantly, CHD was also shown to inhibit the growth of pathogens carrying different tetracycline resistance genes [21,22], whose widespread mechanisms (e.g., efflux, ribosomal protection, degradation, rRNA mutations) hamper the use of tetracyclines as effective therapeutics [23,24]. The in vivo potency of CHD was already described during the 1970s when it was applied in a small phase II clinical study to treat patients suffering from UTIs, including tetracycline-resistant infections [25]. While the antibacterial spectrum of CHD displays certain activity gaps, e.g., against *Pseudomonas* spp. [19], these gaps were further closed by generating the more potent lead molecule CDCHD [15]. The chemical space of both compounds has been further extended in recent studies by biosynthetic engineering and medicinal chemistry approaches to explore the structure–activity relationships of these scaffolds [26,27].

In this study, we investigated in detail the in vitro activity of CHD and CDCHD with emphasis on clinically isolated uropathogens, including multidrug-resistant (MDR) strains. For both compounds, a broad-spectrum activity profile was found with most outstanding activities against MDR *Enterobacteriaceae*, further demonstrating the potential of chelocardins for treatment of UTIs, which are among the most frequent infectious diseases, especially in hospitals, imposing a severe health threat on humans worldwide [29,30]. In parallel, studies on the mechanism of resistance (MoR) revealed even better resistance-breaking properties for CDCHD in comparison to the parent molecule CHD. Development of resistance towards CHD was tested in several bacterial pathogens, but was not detectable except for *K. pneumoniae,* which exhibited an increased expression of the *acrAB-tolC* efflux pump genes triggered by mutations in the repressor gene *ramR*. Intriguingly, while these mutations also induced resistance to several antibiotic classes including tetracyclines, we did not observe any cross-resistance to CDCHD. Indeed, a selection of stable mutants resistant to CDCHD was not feasible under several conditions tested. The absence of evident resistance or very low frequency of resistance (FoR) in pathogens led us to investigate potential self-resistance mechanisms in the natural CHD producer *A. sulphurea.* Moreover, in this case, we only identified an efflux-based resistance gene, which encodes the ChdR exporter associated with the biosynthetic gene cluster of CHD. These findings underline the strong potential of chelocardins, especially the novel lead CDCHD, to be further developed for future antibacterial therapy.

## 2. Results

### 2.1. CHD and CDCHD Were Effective against Clinically Relevant Uropathogens and Displayed Resistance-Breaking Properties In Vitro

To further examine their broad-spectrum activities that were described previously [15,16,19], we tested both chelocardins against extended panels of Gram-positive and Gram-negative bacteria including methicillin-resistant *Staphylococcus aureus* (MRSA) and vancomycin-intermediate *Staphylococcus aureus* (VISA), with the compounds showing antibacterial activity with minimum inhibitory concentrations (MICs) in the low µg/mL range (Table S1). Besides their promising activity against MDR pathogens, both compounds were effective against typical uropathogens among these strains, supporting the potential use of chelocardins to treat UTIs [19,25]. This was further investigated by assaying MICs and minimum bactericidal concentrations (MBCs) of about 100 uropathogenic clinical isolates from a contemporary collection of the DZIF Microbial Genomic Resource Center (MGRC), including various MDR isolates (Table 1; all strains geno- and phenotyped).

The antibacterial activity of both chelocardins was confirmed, and the MIC profiles of these uropathogenic isolates revealed to be similar for CHD and CDCHD. Although CDCHD tended to show slightly increased MICs, which is in contrast to results with non-clinical strains, the new lead molecule displayed a more favorable MBC profile compared to CHD. For selected *K. pneumoniae* and *E. coli* isolates, MIC and MBC values were additionally determined in artificial urine (Table 2).

Compared to values determined in standard test medium, CHD and CDCHD showed ≈4-fold improved antibacterial activities in artificial urine with a concurrent increase of bactericidal activity. The variation of pH between 5.5 and 8.5 in the artificial urine medium revealed generally pH-independent antibacterial activities (referring to MICs), but pH-dependent bactericidal activities with MBCs varying by factor 2–16 (Table S2). Altogether, the bactericidal activity of CDCHD was superior to CHD throughout the whole panel of isolates (except for *Proteus* sp.), and thus its improved potency was confirmed, also tendentially against *P. aeruginosa*, as reported previously [15]. Since Lešnik et al. [15] presumed that differences in susceptibility against *P. aeruginosa* may rely on intrinsic resistance mechanisms such as efflux, we studied the activity of CHD and CDCHD against several efflux-deficient *P. aeruginosa* mutants [31,32,33]. A deletion of the major efflux pump MexAB increased susceptibility of both compounds up to 16-fold, which was also observed for tetracycline (Table S1), whereas deletions of efflux pumps MexCD, MexEF, and MexXY did not lead to increased activities of both compounds. However, since CDCHD is able to inhibit the non-clinical *P. aeruginosa* wild-type strains with 2–8 fold higher potency than CHD and tetracycline, CDCHD may at least partially overcome efflux-based resistance in *P. aeruginosa* for yet unknown reasons. Additionally, the previously reported capability of CHD to inhibit growth of tetracycline-resistant pathogens [21,22] was also shown for CDCHD by testing the susceptibility of strains carrying different sets of common tetracycline resistance determinants including efflux, ribosomal protection, and enzymatic inactivation factors (Table 3). The high potency of CDCHD to inhibit these strains confirmed its wide-ranging tetracycline resistance-breaking properties.

### 2.2. CHD Resistance Development in Target Pathogens Revealed Mutation of ramR in K. pneumoniae

Attempts to raise stable resistant mutants with CHD in *E. coli* or with CDCHD in *K. pneumoniae* and *S. aureus* were generally not successful, most likely due to strong bactericidal effects above threshold concentrations of one- to twofold MIC in the respective strains. In contrast, *K. pneumoniae* treated with increasing concentrations of CHD was susceptible to resistance development. The in vitro exposure to 2 µg/mL CHD led to strongly impaired growth of the wild-type strain *K. pneumoniae* DSM-30104 (Figure S1), and spontaneous resistant mutants occurring at an average frequency of 3.5 × 10^−8^ (FoR) were isolated. Nine independent CHD-resistant mutants were selected and sequentially sub-cultivated until a final concentration of 8 µg/mL CHD was reached. All isolated resistant mutants displayed a significant and stable shift in MIC compared to the parent wild-type strain (Table S3). All mutants showed a normal, exponential growth behavior at 8 µg/mL concentration of CHD, with final cell densities comparable to the wild type (Figure S2), and thus no significant loss in fitness was observed. Cultivation of mutants under non-selective conditions (up to six passages of cultures grown from lag to stationary phase without CHD) did not reveal reversibility of the developed resistance, since a change of MICs was not observed after renewed exposure to CHD.

Comparative whole-genome sequencing of the selected mutants vs. *K. pneumoniae* DSM-30104 wild type revealed, in addition to some inconsistent genomic changes, the regulatory gene *ramR* as the main target of CHD-dependent resistance mutations, which was affected either by nucleotide deletions, insertions, or substitutions (Table S4).

### 2.3. Absence of Cross-Resistance to CDCHD

Extended susceptibility studies of the resistant mutants revealed, in addition to the 4- to 16-fold elevated MICs for CHD compared to *K. pneumoniae* wild type, accompanying co-resistance to chloramphenicol, ciprofloxacin, erythromycin, and several tetracycline derivatives (Table S3). To confirm that decreased susceptibility relies on inactivated *ramR*, as suggested by the sequencing studies, we generated a *K. pneumoniae* DSM-30104 *ramR* knockout mutant (KPΔ*ramR*) using a λ-Red recombinase-based knockout system [34]. Indeed, susceptibility of KPΔ*ramR* towards CHD was decreased by 8- to 16-fold, but only twofold towards CDCHD compared to wild type. Likewise, all tested CHD-resistant mutants showed no significant cross-resistance to CDCHD (Table S5).

### 2.4. Resistance to CHD in K. pneumoniae Was Based on Efflux

The gene product of *ramR* is a repressor of *ramA*, which, in turn, encodes a positive regulator of the AcrAB-TolC efflux system [35,36]. In the context of reduced antibiotic susceptibility, mutations in *ramR* have already been reported to be involved in efflux-mediated resistance to tigecycline in clinical *K. pneumoniae* isolates [37,38]. This led to the assumption that resistance to CHD might also rely on efflux. Thus, we examined the effect of the CHD-induced *ramR* mutations on the regulatory function of the AcrAB-TolC efflux system. In a first experiment, transcription levels of *ramA*, *acrA*, and *acrB* were analyzed by qPCR. As expected, in comparison to the wild type, an upregulation of *ramA* (up to 41-fold) with a concomitant upregulation of *acrA* (up to 12-fold) and *acrB* (up to 67-fold) was observed in all spontaneous *K. pneumoniae*-resistant mutants as well as in KPΔ*ramR* (Table S5). In a second study, eight mutants (Mt8.1–Mt8.8 with genomic changes indicated in Table S4) were subjected to gene expression analysis by RNA-Seq. This transcriptome study revealed that a broader range of regulatory factors was affected in the resistant mutants (Table 4). In addition to *ramRA*, further regulatory cascades involved in controlling *acrAB-tolC* gene expression were differentially expressed, among them the *soxRS* [39,40] and *marRAB* [36,41] operons, showing a strong upregulation of the *soxS* and *marA* activator genes, especially in mutants Mt8.3 and Mt8.6.

This points to a more complex regulatory scenario involving cross-regulation and feedback regulation between and within these operons, which has been shown, for example, for the marbox-dependent activation of *marRAB* expression by a large number of regulators including RamA, SoxS, and MarA [42,43]. On the other hand, most of the mutants showed a downregulation of factors that negatively affect *acrAB-tolC* expression, including the *acrR* repressor gene [36] and the *lon* gene encoding Lon protease, which controls the protein levels of RamA, SoxS, and MarA by degradation [44,45].

To further characterize the major effect of efflux on CHD resistance, we determined MICs by adding the efflux pump inhibitor phenylalanine arginine β-naphthylamide dihydrochloride (PAβN). All *K. pneumoniae* mutants showed both reduced resistance to CHD and reduced co-resistance to tetracycline, tigecycline, chloramphenicol, and ciprofloxacin in the presence of this inhibitor (Table S6), substantially reversing the developed resistance profiles shown in Table S3, while no difference in susceptibility was observed for the wild type. Importantly, PAβN had virtually no effect on the susceptibility towards CDCHD in all strains, demonstrating that its activity remains unaffected by the CHD-mediated resistance mechanism.

### 2.5. ChdR Was Found to be the Primary Self-Resistance Factor in Natural CHD Producer A. sulphurea

Upon identification of the CHD biosynthetic gene cluster [46], the *chdR* gene encoding a putative multidrug efflux resistance protein from the EmrB/QacA subfamily was hypothesized to provide a mechanism of self-resistance to *A. sulphurea* by serving as an exporter of the antibiotic. To test this hypothesis, a cosmid library of the *A. sulphurea* genome was prepared and expressed in *A. mediterranei*, a related species devoid of the CHD biosynthetic gene cluster and susceptible to CHD. It was found that only *A. sulphurea* genomic fragments carrying the *chdR* gene were able to confer resistance to CHD in *A. mediterranei*.

Another attempt to identify further possible CHD resistance factors in the native producer was made by generating an *A. sulphurea* Δ*chdPKS* Δ*chdAR* double mutant devoid of the essential CHD biosynthetic genes *chdPKS* (encoding the minimal polyketide synthase) and the proposed resistance cassette *chdAR* (encoding ChdR and its regulator ChdA). The selected double mutant showed an MIC for CHD of 2.5 μg/mL, in contrast to the parent strain *A. sulphurea* Δ*chdPKS* growing in the presence of >100 μg/mL of CHD [46], and was subjected to development of CHD-resistant mutants. Indeed, several resistant mutants carrying the Δ*chdPKS* Δ*chdAR* background were generated at CHD concentrations of 20–30 µg/mL (8- to 12-fold MIC), and a set of nine mutants was selected for further analysis (Table S7). A comparative whole-genome sequencing of reference strain vs. selected mutants revealed that all of the mutants carried single-point mutations within one specific target gene encoding the AfsR/SARP regulator (Table S8). The mutated regulator gene, a close homolog to the daunorubicin and doxorubicin biosynthesis and efflux master regulator DnrI in *Streptomyces* [47], locates next to genes of *A. sulphurea* that encode a homolog of the multidrug ABC transporter DrrAB, which is the daunorubicin efflux pump in *Streptomyces peucetius* [48].

To test if an overexpression of the *drrAB* genes of *A. sulphurea* conferred CHD resistance to the *A. sulphurea* Δ*chdPKS* Δ*chdAR* double mutant, constructs for expression of either *drrAB* or *chdR* (under the control of strong promoters P*_actI_* and P_ermE*_, respectively) or *chdAR* (under control of the native promoter) were generated and used for transformation of the double mutant. However, while transformants expressing *chdR* or *chdAR* showed resistance towards both CHD and CDCHD similar to *A. sulphurea* wild type [15,46], overexpression of *drrAB* did not lead to an increase in resistance, suggesting that *drrAB* genes alone are not sufficient to mediate resistance to chelocardins through efflux, and that the AfsR/SARP regulator of *A. sulphurea* might control further (yet unknown) genes involved in an alternative resistance-conferring route. In conclusion, ChdR was identified here as the primary efflux-based resistance determinant in the natural producer that accepts both CHD and CDCHD as substrates.

## 3. Discussion

The drop in effectiveness of clinically important antibiotic classes, based on rapidly spreading resistance among a variety of bacterial species, illustrates the urgent need of alternative treatments [1,49,50]. Due to a shortage in the discovery of new effective molecules, an alternative strategy deals with the optimization of already known but neglected scaffolds. For CHD, structural modifications introduced through genetic engineering led to the amidated derivative CDCHD exhibiting improved pharmaceutical properties and an extended antibacterial spectrum [15]. In this study, we confirmed the enhanced activity pattern for CDCHD within the ESKAPE pathogen panel and, additionally, its activity against tetracycline-resistant pathogens. An early report on successful CHD therapy of patients suffering from pyelonephritis [25] led us to reassess CHD as a scaffold for the development of a novel therapeutic against UTIs. Here, we consolidated the prospect of treating this infectious disease by using CHD-based novel antibacterial scaffolds. The in vitro activity of both CHD and CDCHD was demonstrated on a large panel of uropathogens from a recent clinical isolate collection, including colistin-resistant pathogens and pathogens expressing TEM-β lactamases or extended-spectrum β-lactamases. The highest antibacterial potency of the compounds was observed against uropathogenic *Enterobacteriaceae*, which represent about 70–80% of all common uropathogens causing either uncomplicated or complicated UTIs [30]. The fact that both chelocardins show even better activities when MICs were determined in artificial urine furthermore underlines their potential as future UTI drugs, and MBCs were generally lower for CDCHD against most of the clinical uropathogens both in standard medium and in artificial urine, demonstrating the advanced potency of this molecule. The fact that a general loss in activity was observed against the uropathogenic isolates of *P. aeruginosa* compared to previously reported MICs for non-uropathogenic clinical isolates of *P. aeruginosa* [15] suggests that the uropathogenic strains may possess additional resistance determinants yet to be identified. However, *P. aeruginosa* plays a minor role in the development of different forms of UTI, sharing a fraction of 1–2% among epidemiologically relevant uropathogens [30]. Overall, the activity profiles of chelocardins are highly remarkable because conventional drugs used to treat UTIs, such as fluoroquinolones or β-lactam antibiotics, are affected by the increasing number of MDR uropathogens that hamper their application [51,52,53,54]. In addition, the use of fluoroquinolones including those commonly applied in UTI therapy such as ciprofloxacin has recently been restricted due to severe side effects [55,56], making the development of novel drugs for UTI treatment even more urgent.

**Figure 2:**
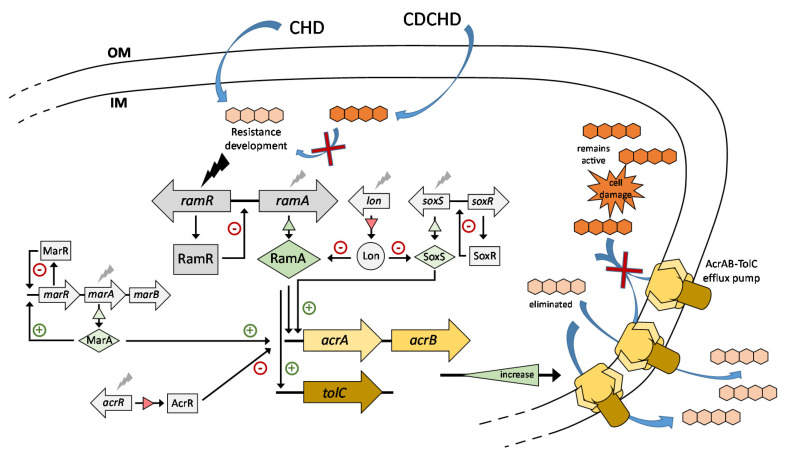
Current model of genes and regulatory cascades affected during resistance development against CHD in *K. pneumoniae*, leading to dysregulation of efflux mediated by AcrAB-TolC (pump schematically presented according to structural models [57,58]). In contrast to CHD, CDCHD does not lead to the development of resistant mutants and it is furthermore able to escape elimination through efflux (red crosses), hence retaining antimicrobial activity. Bolt-shaped arrows indicate resistance-associated effects either by direct mutation in the case of *ramR* (black bolt) or change of expression levels of various factors controlling *acrAB-tolC* expression (grey bolts). Gene products in grey rectangles and green diamonds represent transcriptional repressors and activators, respectively. Green and red triangles (associated with black arrows) represent increasing and decreasing amounts of gene products with direct influence on AcrAB-TolC production, respectively. The genes or operons affected by CHD-mediated resistance include *ram* (resistance antibiotic multiple), *sox* (sulfur-oxidizing operon), *mar* (multiple antibiotic resistance), *lon* (encoding a serine protease), and *acrAB-tolC* (encoding the antibiotic efflux pump) [36,41]. OM, outer membrane; IM, inner membrane.

The potential of CDCHD as a novel antibacterial lead was further demonstrated by revealing its resistance-breaking properties, not only towards a diverse set of tetracycline-resistant determinants, but also by overcoming the efflux-based resistance induced by the parent compound CHD itself. Our studies on a set of CHD-resistant mutants showed no apparent cross-resistance to CDCHD, and several attempts to induce resistance towards CDCHD by using different Gram-positive and Gram-negative bacterial species did not lead to the development of any stable CDCHD-resistant mutant in vitro. While resistance towards CHD was found to rely mainly on the upregulation of the ubiquitous AcrAB-TolC multidrug efflux pump, the potency of CDCHD was generally not affected by this mechanism, thus indicating that CDCHD might be a weak substrate for AcrAB-mediated efflux and does not select for its deregulation (Figure 2).

This is particularly important because therapy with newly approved tetracycline derivatives is already affected by the emergence of resistant bacteria. The semisynthetic minocycline derivative tigecycline is the first FDA-approved member of the family of glycylcyclines [59], which was initially developed to overcome resistance against classical tetracyclines [60,61]. However, cases of tigecycline-resistant bacteria have been reported for several years, which are augmented by recent studies showing that ribosome mutations and the spread of tetracycline-inactivating enzymes affect the efficacy of further third generation tetracyclines including omadacycline and eravacycline [23,62,63]. Remarkably, an important resistance mechanism for tigecycline relies on mutations of *ramR* and, consequently, RamA-mediated upregulation of the AcrAB-TolC efflux pump [38,64,65]. Further efflux-based mechanisms of resistance towards omadacycline and eravacycline have been recently reported [66,67,68,69]. Moreover, in the case of resistance development against (fluoro)quinolones or β-lactams such as cefoxitin, mutations in *ramR* were shown to be the primary and most frequent cause for efflux-mediated MDR phenotypes in species such as *Salmonella enterica* or *K. pneumoniae* [35,70]. Furthermore, clinical isolates with different resistance-conferring mutations and phenotypes were often found to overexpress additional positive regulators of *acrAB-tolC* such as MarA and SoxS [71,72,73], which was also observed in this study. As CDCHD did not induce any of these resistance mechanisms and is not affected by them, we can conclude that this compound might eventually be more potent than currently applied antibiotics, especially for the treatment of infections caused by MDR bacteria, since resistance-breaking properties of CDCHD have also been demonstrated in a concise set of clinically relevant uropathogenic strains as discussed above. However, the compound’s future development will also hinge on evaluating its potency using larger sets of MDR bacteria with a particular focus on strains that display an upregulated efflux or possess clinically relevant efflux determinants that were not yet included in recent screenings.

Our study further showed that the only factor identified thus far to confer resistance towards both CHD and CDCHD is the ChdR exporter of the natural CHD producer *A. sulphurea*, which is restricted to the presence of the CHD biosynthetic gene cluster in this soil-dwelling bacterium. Altogether, CDCHD overcomes resistance mechanisms exerted not only on various tetracyclines but also on the parent molecule CHD, and the antibacterial spectrum of CDCHD and in particular its potent activity against clinical uropathogens make the compound an ideal candidate for in vivo efficacy testing. Currently, its pharmacokinetic and pharmacodynamic properties are investigated in mouse and rat studies including (c)UTI models with selected uropathogens, aiming to develop the lead molecule further towards a novel broad-spectrum antibiotic.

## 4. Materials and Methods

### 4.1. Chemicals

Chelocardin and 2-carboxamido-2-deacetyl-chelocardin were isolated as described previously [15,46]. Both compounds were prepared as sodium salts with citrate as stabilizer, as described previously [74], and stock solutions were prepared at 1 mg/mL in demineralized water. All tetracycline analogs as well as rifampicin, erythromycin, polymyxin B, chloramphenicol, ciprofloxacin, and vancomycin were obtained from Sigma Chemicals Co., St. Louis, MO, USA. Kanamycin and ampicillin were from Carl Roth GmbH, Karlsruhe, Germany.

### 4.2. Antimicrobial Screening

All microorganisms were handled according to standard procedures and were obtained from the German Collection of Microorganisms and Cell Cultures (Deutsche Sammlung von Mikroorganismen und Zellkulturen, DSMZ) and the American Type Culture Collection (ATCC), or were part of our internal strain collection or provided by collaboration partners. MICs for *M. bovis* were determined in 5 mL cultures (3 to 4 weeks incubation) and all other values were determined by micro-dilution assays [75]. Overnight cultures were diluted in modified tryptic soy broth (TSB; from BD, Heidelberg, Germany; used for *Enterococcus* spp., *Amycolatopsis* sp.), in Middlebrook 7H9 (M7H9; Sigma-Aldrich, Munich, Germany; used for mycobacteria) or cation-adjusted Mueller–Hinton broth (CAMHB; from BD, Heidelberg, Germany; used for all other listed bacteria) to achieve a final inoculum of approximately 1 × 10^6^ CFU/mL. Serial dilutions of compounds were prepared in sterile clear 96-well plates. The cell suspension was added, and microorganisms were grown for 16–18 h at either 30 °C or 37 °C (*Enterococcus* spp. were grown under microaerophilic conditions). Growth inhibition was assessed by visual inspection and MIC values were determined as the lowest concentration of antibiotic at which no visible growth was observed.

MIC determinations of clinical bacterial uropathogens were performed by micro-dilution assays according to guidelines of the European Committee on Antimicrobial Susceptibility Testing [76]. MIC values were determined as lowest concentration leading to no visible growth. Minimum bactericidal concentrations (MBC) were determined by inoculation of 3 μL/well of the 96-well plates (used for MIC determination) to rectangular blood-agar plates (Iso-Sensitest + 5% sheep blood) and incubated overnight at 37 °C. MBC values were determined as the lowest concentration that leads to killing of at least 99.9% CFU of each inoculum. Selected uropathogens were analyzed in artificial urine medium prepared as described previously [77], which was adjusted to different pH values (5.5–8.5).

### 4.3. In Vitro Selection of CHD-Resistant Klebsiella Mutants

Chelocardin-resistant mutants were generated by exposing wild-type strain *K. pneumoniae* DSM-30104 to stepwise increasing CHD concentrations (0, 2, 4, 8 μg/mL) using a repetitive procedure of inoculation from agar plates into liquid medium with a concurrent increase of CHD concentration. Genomic DNA of all mutants was prepared by resuspending bacteria from 5 mL overnight cultures in 1.8 mL 10 mM Tris-HCl (pH 8.0) and incubation of all samples for 3.5 h at 37 °C. A total of 200 μL proteinase K (20 mg/mL) and 20 μL 20% (*w*/*v*) SDS were added and incubated for 2 h at 55 °C. Then, 100 μL RNase A (20 mg/mL) was added and incubated for an additional 30 min at 37 °C. DNA was extracted using the standard phenol-chloroform method, followed by addition of 1/10 volume of 3 M NaOAc (pH 4.8) and 2.5 volumes of cold EtOH. DNA was spooled out, washed with 70% EtOH, and diluted in deionized water at 55 °C and 300 rpm for 1 h. DNA concentration and purity were measured by using a NanoDrop spectrometer. Fitness costs of CHD-resistant mutants were determined by diluting overnight cultures to a final optical density at 600 nm of 0.05. Bacterial growth was monitored over 24 h by a continuously measuring the optical density at 600 nm. Growth curves were obtained by plotting optical density versus time.

### 4.4. Construction of a K. pneumoniae ramR Deletion Mutant

The deletion of *ramR* in *K. pneumoniae* DSM-30104 was performed by using a λ-Red recombinase-based knockout system, as described by Huang et al. [34]. Primer pairs for generating the deletion construct (RamRKO_fwd/RamRKO_rev) and for confirmation of the *ramR* knockout (RamRconf_fwd/RamRconf_rev) are listed in Table S9.

### 4.5. Construction of A. sulphurea Double Mutant and Complementation Vectors

The *chdAR* cassette (encoding efflux pump ChdR, its regulator ChdA, and an operator/promoter region) was deleted from *A. sulphurea* Δ*chdPKS* mutant [46], which does not produce CHD. For that purpose, two 1 kb homology cassettes for deletion of *chdAR* via homologous recombination (primer pairs chdARLF/chdARLR and chdARRF/chdARRR listed in Table S9) were cloned via *Sph*I and *Eco*RI into plasmid pNV18 [78], which was transformed into *A. sulphurea* Δ*chdPKS* according to previously described protocols [26,46]. In a selected transformant, the deletion of *chdAR* was identified by colony PCR. For complementation with *chdR*, *chdAR*, and *drrAB* genes, amplified fragments (primer pairs chdRF/chdRR, chdAR/chdRR, and DrrAF/DrrBR, respectively, listed in Table S9) were cloned via *Nde*I and *Xba*I into plasmid vectors to generate constructs pAB03e*chdR, pAB03(ΔPactI)chdAR, and pAB03drrAB, respectively, which were transformed into the double mutant. The derivatives of vector pAB03 [46] are pAB03e*, in which the *actII*-ORF4/P*_actI_* promoter of the pAB03 vector is replaced by modified promoter P_ermE__*_ from *Saccharopolyspora erythraea* (source AciesBio), and pAB03(ΔPactI) that does not carry the *actII*-ORF4/P*_actI_* activator/promoter system [79]. To check for increased resistance against CHD or CDCHD, we grew transformants on TSB agar plates with CHD or CDCHD gradient (0–10 μg/mL).

### 4.6. In Vitro Selection of CHD-Resistant Amycolatopsis Mutants

The *A. sulphurea* Δ*chdPKS* Δ*chdAR* double mutant (MIC 2.5 μg/mL) was first grown in liquid TSB until dense growth and then supplemented with 10 μg/mL of CHD. After 1 day, the culture was plated on TSB agar with the same antibiotic concentration, and single colonies were selected for inoculation into liquid TSB medium supplemented with 15 μg/mL of CHD. The procedure was further repeated, and single colonies were inoculated into TSB medium supplemented with either 20 μg/mL or 30 μg/mL of CHD. Finally, five mutants developed at 20 μg/mL CHD and four mutants developed at 30 μg/mL CHD was selected for isolation of genomic DNA according to published protocols [46] together with the reference strain.

### 4.7. Comparative Whole-Genome Sequencing

Whole-genome sequencing (WGS) of *K. pneumoniae* DSM-30104 wild type and CHD-resistant mutants was performed using Illumina sequencing technology on a MiSeq platform at the Helmholtz Centre for Infection Research (Braunschweig, Germany). Sequencing was performed in paired-end fashion. Raw data were analyzed by an alignment against the reference genome sequences of *K. pneumoniae* DSM-30104. The genome sequence of *K. pneumoniae* DSM-30104 was not readily available from the public databases; therefore, to obtain its complete reference sequence, we submitted high-molecular-weight DNA for sequencing with PacBio technology at the German Collection of Microorganisms and Cell Cultures (DSMZ). After de novo sequence assembly of raw data with SMRT portal provided by Pacific Biosciences (Menlo Park, CA, USA), we obtained a single contig for each chromosome and three extrachromosomal replicating elements (chromosome: 5,295,933 bp; pKPD1: 105,910 bp; pKPD2: 96,086 bp; pKPD3: 44,026 bp). The reference-guided alignment of Illumina raw sequencing data for all mutant samples was carried out in Geneious software (Geneious Prime 2019.0.4) [80] with “low sensitivity” parameters, otherwise the parameters were left default. The consensus calling for mutant and control strains was performed in Geneious software by executing the “generate consensus sequence” command and using “highest quality” as consensus calling algorithm, while other parameters were left default. Comparison of consensus sequences generated in the previous step was performed in Geneious software with the help of “MAUVE” whole-genome alignment plugin. Apart from using default settings, the “assume collinear genomes” option was selected to be on. The final step of the analysis comprised the manual verification of consensus inconsistencies between samples and comparing those to the reference and control sample sequences.

The same procedure was applied for WGS of *A. sulphurea* NRRL 2822 wild type, *A. sulphurea* Δ*chdPKS* Δ*chdAR* double mutant, and *A. sulphurea* CHD-resistant mutants, with the following differences: Raw data were analyzed by an alignment against the reference genome sequences of *A. sulphurea* NRRL 2822. The genome sequence of *A. sulphurea* NRRL 2822 was not readily available from the public databases; therefore, to obtain its reference sequence, we submitted high-molecular-weight DNA for sequencing with Illumina technology at the Helmholtz Centre for Infection Research (Braunschweig, Germany). After de novo sequence assembly of a combination of paired-end and mate-pair reads with ABySS software [81], we obtained a set of 8 contigs (N50: 2,197,275 bp). The reference-guided read alignment and the following analysis steps were identical.

### 4.8. Quantification of Gene Expression by qPCR

Total RNA of 150 mL bacterial cultures of *K. pneumoniae* DSM-30104 wild type and CHD-resistant mutants was isolated using the miRNeasy mini kit (Qiagen, Hilden, Germany). Concentration and purity of RNA was measured by using a Nanodrop spectrometer. cDNA was synthesized using the RevertAid Premium Reverse Transcriptase (ThermoScientific, Schwerte, Germany) following the manufacturer’s instructions. Gene expression was measured by qPCR using the Peqstar 96Q (Peqlab, Erlangen, Germany) cycler. cDNA was diluted 1:10 and primer pairs listed in Table S10 were used. Target genes were amplified using the GoTaq Master Mix (Promega, Madison, WI, USA), which includes a green dye for detection of dsDNA, in 10 µL reaction mixtures, as described by the manufacturer. Samples were measured in triplicate and relative transcription levels compared to the wild type were calculated by the 2^−ΔΔCt^ method [82] and normalized to 16S rRNA.

### 4.9. Gene Expression Analysis by RNA-Seq

Independent duplicate cultures of *K. pneumoniae* DSM-30104 wild type and CHD-resistant mutants (Mt8.1–Mt8.8) were inoculated to a starting OD_600_ of <0.01 and grown without selective pressure for 18 h each in CAMHB medium (to final ODs of 3.5–4.5). Cells were collected at 4 °C and pellets were frozen in liquid nitrogen. For total RNA isolation, we suspended cell pellets in 600 µL of lysis buffer (0.5 mg/mL lysozyme, TE pH 8.0), prior to the addition of 60 µL 10% w/v SDS and inverting the tubes. The mixtures were heated for 2 min to 64 °C in a water bath, prior to the addition of 66 µL 3M NaOAc (pH 5.2) and 1 M EDTA, and mixing by inversion. Then, each 750 µL phenol (Roti-Aqua phenol, # A980.3; Carl Roth GmbH, Karlsruhe, Germany) was added, the tubes were inverted, and the mixture was incubated again for 6 min in the 64 °C water bath. Afterwards, samples were put on ice, centrifuged (15 min, 13,000 rpm, 4 °C), and the aqueous phases were transferred to 2 mL Phase Lock Gel (PLG) tubes (QuantaBio, Beverly, MA, USA). After each addition of 750 µL of chloroform, we mixed the tubes vigorously for 20 s, centrifuged (12 min, 13,000 rpm, 4 °C) them, and then transferred the aqueous layers to new 2 mL tubes. After we added each 1.4 mL of 30:1 mixes (EtOH:3M NaOac, pH 6.5), RNA was precipitated at −20 °C overnight, and the pellet was washed once in 75% v/v ethanol, air-dried, and resuspended in 50 µL pre-warmed (65 °C) water. To remove genomic DNA, we incubated RNA samples with each 0.25 U of DNase I (Fermentas, Vilnius, Lithuania) per 1 µg of RNA for 45 min at 37 °C and performed efficient removal confirmed by control PCR. cDNA libraries were generated at Vertis Biotechnologie AG (https://www.vertis-biotech.com; Freising-Weihenstephan, Germany), deliberately omitting an rRNA depletion step as follows. Total RNA was sheared via ultrasound sonication (four 30-s pulses at 4 °C) to generate on average 200- to 400-nt fragments. Fragments of <20 nt were removed using the Agencourt RNAClean XP kit (Beckman Coulter Genomics, Indianapolis, IN, USA) and the Illumina TruSeq adapter was ligated to the 3′ ends of the remaining fragments. First-strand cDNA synthesis was performed using M-MLV reverse transcriptase (NEB, Ipswich, MA, USA), wherein the 3′ adapter served as a primer. The first-strand cDNA was purified, and the 5′ Illumina TruSeq sequencing adapter was ligated to the 3′ end of the antisense cDNA. The resulting cDNA was amplified by PCR to about 10 to 20 ng/µL using a high-fidelity DNA polymerase. The TruSeq barcode sequences were part of the 5′ and 3′ TruSeq sequencing adapters. The cDNA library was purified using the Agencourt AMPure XP kit (Beckman Coulter Genomics, Indianapolis, IN, USA) and analyzed by capillary electrophoresis (Shimadzu MultiNA microchip; Shimadzu Corporation, Kyoto, Japan). Libraries were sequenced on a NextSeq 500 platform in single-read mode with a read length of 75 bp. The raw sequencing data were aligned to the complete genome sequence of *K. pneumoniae* DSM-30104 in Geneious reference-based assembler. Reads were subsequently counted against coding sequence (CDS) features, and those ambiguously mapped were considered partial (0.5) matches. Obtained read counts were subjected to differential transcript level analysis in DESeq2 package in R programming language.

### 4.10. Preparation of an A. sulphurea Genomic Cosmid Library and Its Expression in A. mediterranei

The genomic DNA from *A. sulphurea* NRRL 2822 (ARS Culture Collection) was isolated partially digested with *Sau*3AI, and the obtained DNA fragments with an approximate size of 35–40 kb were ligated into the *Bam*HI site of replicative conjugative cosmid vector pOJ456, a modified version of the pOJ436 vector [83], where 2.5 kb ΦC31 integrase cassette was excised with *Hin*dIII (overhangs were filled with Klenow polymerase) and replaced with 2.5 kb pSG5 replication cassette (encoding the rolling circle replication initiator protein Rep [84]) excised with *Eco*81I and *Sph*I (overhangs were filled with Klenow polymerase) from medium copy number vector pKC1139 [83]. The ligated DNA was packaged into phage particles using Gigapack III Gold Packaging kit (Agilent Technologies, Santa Clara, CA, USA), and phages were used for transduction of non-methylating *E. coli* GB2006. Titering experiments were performed to achieve the best conditions to grow single colonies, and the library was then amplified by using large agar plates. Clones from all plates were collected, mixed, and grown in a larger liquid culture to isolate the cosmid library DNA by using the GeneJet Miniprep kit (ThermoScientific, Schwerte, Germany). The cosmid library DNA was chemically transformed into *A. mediterranei* CBS121.63 (CBS Culture Collection) [85], and the transformants were selected with apramycin (resistance cassette encoded on the cosmid). More than 1000 selected transformants (at least 5x coverage of the *A. sulphurea* genome) were grown on TSB agar plates with different CHD gradients (between 0 and 30 µg/mL) to identify the cosmids expressing CHD resistance gene(s) by either sequencing or colony PCR.

## Figures and Tables

**Table 1:** Minimal inhibitory concentrations (MICs) and minimum bactericidal concentrations (MBCs) of CHD and CDCHD against uropathogenic clinical isolates grown in cation-adjusted Mueller–Hinton broth (CAMHB).

Isolate (*n* = Number of Strains)	MIC (µg/mL)		MBC (µg/mL)	
	CHD	CDCHD	CHD	CDCHD
Escherichia coli *				
Sensitive (15)	2	4	16	4
TEM β-lactamase (8)	2–4	8	16	8
ESBL (7)	4	4	32	8
Colistin-resistant (5)	8	8	32	16
*Enterobacter* spp. * (8)	4	4	16	8–16
*Klebsiella* spp. *				
Sensitive (7)	4	4	16	8
ESBL (2)	4	4–8	32	8–16
Carbapenem-resistant (2)	4	4–8	32–64	8
*Enterococcus faecalis* (19)	8	16	>64	16
*Enterococcus faecium* (6)	4	8	32	16
*Proteus* spp.				
Sensitive (7)	4	8	8–16	64
ESBL (2)	4	8–32	8–16	16–32
*Pseudomonas aeruginosa* (10)	>64	32–64	>64	>64

For each group of strains, median values determined in three independent measurements per isolate are shown. ESBL, extended-spectrum β-lactamases. * Pathogens belonging to the family of *Enterobacteriaceae*.

**Table 2:** Minimal inhibitory concentrations (MICs) and minimum bactericidal concentrations (MBCs) of CHD and CDCHD determined for 10 clinical isolates (selected from strain panel shown in Table 1) in artificial urine (pH 6.5) and standard CAMHB medium. Values denote medians of three independent measurements per isolate.

Test Condition	Isolate (*n*=)	MIC (µg/mL)		MBC (µg/mL)	
		CHD	CDCHD	CHD	CDCHD
Artificial urine	*Escherichia coli* (7)	1	1	8	2
	*Klebsiella pneumoniae* (3)	1	1	8	4
CAMHB	*Escherichia coli* (7)	4	4	32	8
	*Klebsiella pneumoniae* (3)	4	4	32	8

**Table 3:** Minimal inhibitory concentrations (MICs) of CDCHD and tetracycline (TET) against strains carrying different tetracycline resistance determinants.

TET-Resistant Strain	Resistance Gene	Resistance Mechanism	MIC (µg/mL)	
			CDCHD	TET
*E. coli*	*tetB*	Efflux	4	>64
*E. coli*	*tetM*	Ribosomal protection	0.5	64
*E. coli*	*tetW*	Ribosomal protection	1	>64
*E. coli* 49	*tetB*	Efflux	4	>64
*E. coli* 74	*tetB*	Efflux	8	>64
*Serratia liquefaciens*	*tetB*; *tet34*	Efflux; enzymatic inactivation	2	>64
*Pseudomonas pseudoalcaligenes*	*tetB*; *tet34*	Efflux; enzymatic inactivation	2	>64
*K. pneumoniae* 3	*tetA*	Efflux	8	>64
*K. pneumoniae* 8	*tetA*	Efflux	32	>64
*K. pneumoniae* 24	*tetA*	Efflux	8	>64

**Table 4:** RNA-Seq analysis of differential gene expression in *K. pneumoniae* CHD-resistant mutants (Mt8.1–Mt8.8). Genes are grouped according to the annotated function (efflux component, regulator or protease) of respective gene products, and values are given as fold-changes of expression levels (on the basis of reads per kilobase of transcript, per million mapped reads; RPKM) relative to the parent strain (*K. pneumoniae* DSM-30104 wild type).

Gene	Mt8.1	Mt8.2	Mt8.3	Mt8.4	Mt8.5	Mt8.6	Mt8.7	Mt8.8
**Efflux**								
*acrA*	2.65	1.72	1.97	2.16	2.03	2.33	1.98	2.88
*acrB*	3.27	2.82	2.01	2.70	2.88	2.48	3.03	2.31
*tolC*	2.97	2.00	2.25	2.15	2.10	3.39	2.12	3.96
**Regulators**								
*ramR*	5.80	4.44	4.35	6.78	4.89	5.59	4.98	3.01
*ramA*	16.57	11.79	10.47	13.40	9.89	13.73	10.91	6.11
*soxR*	1.02	1.03	3.22	0.70	0.93	3.83	0.81	0.73
*soxS*	1.13	1.09	18.09	0.81	1.12	16.80	0.86	0.91
*marR*	5.22	1.49	17.79	4.16	1.13	20.16	1.11	2.93
*marA*	2.58	1.14	11.69	2.65	0.83	11.66	0.75	0.78
*marB*	2.84	1.71	17.47	3.86	1.23	13.69	1.30	1.30
*acrR*	0.63	1.09	1.01	0.67	0.95	0.76	1.10	0.41
**Protease**								
*lon*	0.78	0.86	0.28	0.76	0.87	0.35	0.84	0.92

## Supplementary Materials

The following are available online at https://www.mdpi.com/2079-6382/9/9/619/s1, Figure S1: Time-kill curves of *K. pneumoniae* DSM-30104 exposed to CHD (MIC: 1 μg/mL) and CDCHD (MIC: 0.5 μg/mL) at 0.5- and 2-fold MIC. Cell viability was determined over 24 h by three independent CFU (colony forming units) counts. Data are represented as mean values ± standard deviation, Figure S2: Growth curves of *K. pneumoniae* DSM-30104 wild type and CHD-resistant mutants (Mt8.1–Mt8.10) over 24 h. Data represent OD_600_ values at each time point. The nine mutant strains were cultivated in presence of 8 μg/mL CHD (= 8-fold MIC), Table S1: MIC values of CHD, CDCHD and tetracycline (TET) for Gram-positive and Gram-negative bacteria (non-clinical strains). ^a^ multidrug-resistant *S. aureus*; ^b^ methicillin-83 resistant *S. aureus*; ^c^ vancomycin-intermediate *S. aureus*; n.d.: not determined, Table S2: Minimal inhibitory concentrations (MICs) and minimum bactericidal concentrations (MBCs) of CHD and CDCHD for *E. coli* and *K. pneumoniae* clinical isolates determined in artificial urine at different pH values. Values denote median of three independent measurements per isolate, Table S3: Susceptibility of *K. pneumoniae* DSM-30104 wild type (Wt) and *K. pneumoniae* CHD-resistant mutants (Mt8.1–Mt8.10) to various antibiotics, Table S4: Mutations identified in *K. pneumoniae* DSM-30104 CHD-resistant mutants (Mt8.1–Mt8.10) by whole genome sequencing. bp: base pair; Ins: insertion; Δ: deletion; #: number of affected bp; RE: repeat expansion. Change of codon function indicated by respective amino acids (in one letter code), Table S5: Relative transcription levels of *ramA*, *acrA* and *acrB* genes of *K. pneumoniae* CHD-resistant mutants in comparison to *K. pneumoniae* DSM-30104 wild type (analyzed by qPCR). n.d.: not determined, Table S6: Activity of *K. pneumoniae* DSM-30104 wild type and CHD-resistant mutants in the presence of phenylalanine arginine β-naphthylamide dihydrochloride (PAβN). TET: tetracycline; TIG: tigecycline; CM: chloramphenicol; CIP: ciprofloxacin, Table S7: MIC values of CHD-resistant mutants (selected strains C2087–C2100) developed from *A. sulphurea* Δ*chdPKS* Δ*chdAR* (parent strain); determined in TSB medium, Table S8: Mutations identified in *A. sulphurea* Δ*chdPKS* Δ*chdAR* CHD-resistant mutants (strains C2087–C2100, see Table S7) by whole genome sequencing. Change of codon function indicated by codon number and respective amino acids (in one letter code), Table S9: Primers for *K. pneumoniae ramR* and *A. sulphurea chdAR* gene deletions and vector constructs, Table S10: Primers used for qPCR.
