# Supplementary material for: Amidochelocardin Overcomes Resistance Mechanisms Exerted on Tetracyclines and Natural Chelocardin
Source: Antibiotics (Basel). 2020 Sep 18;9(9):619. doi: 10.3390/antibiotics9090619 (PMC7559539; doi:10.3390/antibiotics9090619)
Supplement: Supplementary file 1 [file antibiotics-09-00619-s001.pdf]

Amidochelocardin overcomes resistance mechanisms exerted on tetracyclines and natural chelocardin

Supplementary Materials

Fabienne Hennessen[1,2,†], Marcus Miethke[1,2,†], Nestor Zaburannyi[1,2], Maria Loose[3], Tadeja Lukežič[1,2,4], Steffen Bernecker[2,5], Stephan Hüttel[2,5], Rolf Jansen[2,5], Judith Schmiedel[6], Moritz Fritzenwanker[6], Can Imirzalioglu[6], Jörg Vogel[7], Alexander J. Westermann[7], Thomas Hesterkamp[2], Marc Stadler[2,5], Florian Wagenlehner[3], Hrvoje Petković[8], Jennifer Herrmann[1,2,*] and Rolf Müller[1,2,*]

[1]Department of Microbial Natural Products, Helmholtz Institute for Pharmaceutical Research Saarland (HIPS) - Helmholtz Centre for Infection Research (HZI), and Department of Pharmacy, Saarland University Campus E8.1, 66123 Saarbrücken, Germany

[2]German Center for Infection Research (DZIF), Partner Site Hannover-Braunschweig, 38124 Braunschweig, Germany

[3]Clinic for Urology, Paediatric Urology & Andrology, Justus-Liebig University Gießen, 35392 Gießen, Germany, and German Center for Infection Research (DZIF), Partner Site Giessen-Marburg-Langen

[4]National Institute of Biology, Večna pot 111, 1000 Ljubljana, Slovenia

[5]Department of Microbial Drugs, Helmholtz Centre for Infection Research (HZI), Inhoffenstrasse 7, 38124 Braunschweig, Germany

[6]Institute of Medical Microbiology, Justus-Liebig University Gießen, Germany, 35390 Gießen, Germany, and German Center for Infection Research (DZIF), Partner Site Giessen-Marburg-Langen

[7]Helmholtz Institute for RNA-based Infection Research (HIRI), Helmholtz Centre for Infection Research (HZI) and Institute of Molecular Infection Biology (IMIB), University of Würzburg, Josef-Schneider-Str. 2, 97080 Würzburg, Germany

[8]Department of Food Science and Technology, Biotechnical Faculty, University of Ljubljana, Jamnikarjeva 101, 1000 Ljubljana, Slovenia

† These authors contributed equally to this study.

* Corresponding authors:

jennifer.herrmann@helmholtz-hips.de, Tel.: +49 681 98806 3101

rolf.mueller@helmholtz-hips.de, Tel.: +49 681 98806 3000

Supplementary Figures

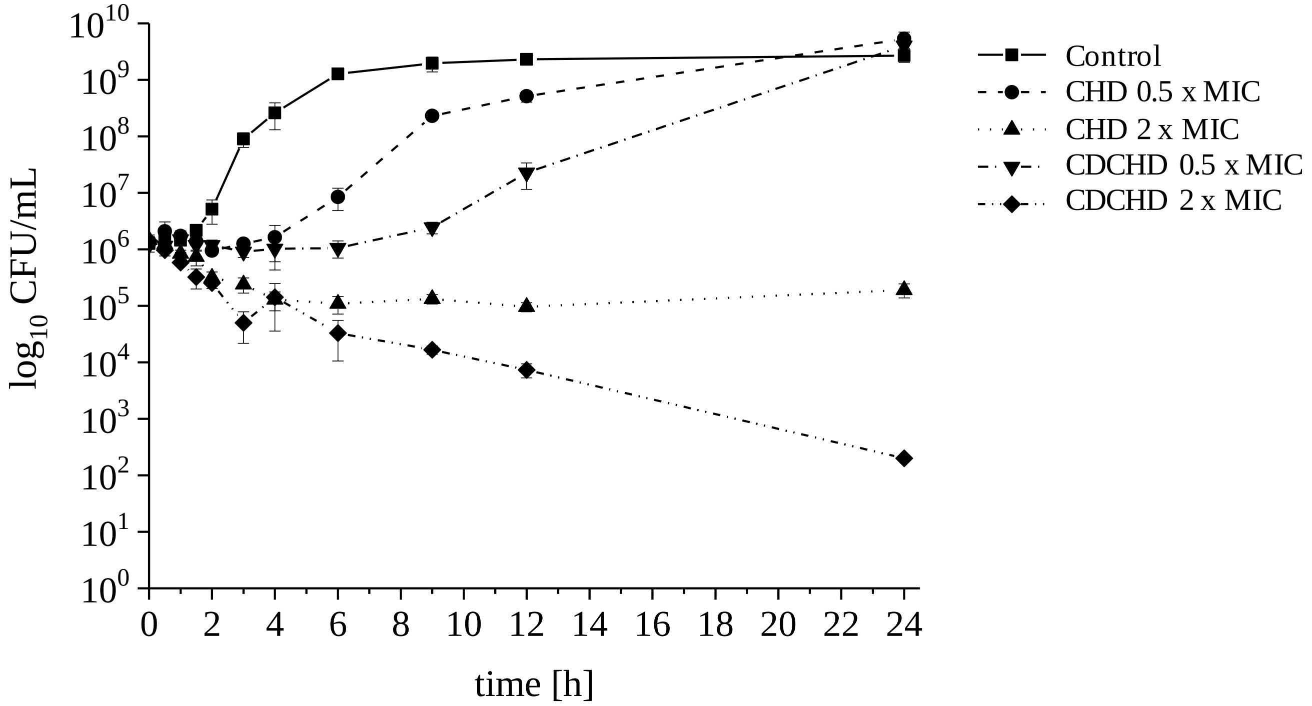

Figure S1. Time-kill curves of *K. pneumoniae* DSM-30104 exposed to CHD (MIC: 1 µg/mL) and CDCHD (MIC: 0.5 µg/mL) at 0.5- and 2-fold MIC. Cell viability was determined over 24 h by three independent CFU (colony forming units) counts. Data are represented as mean values ± standard deviation.

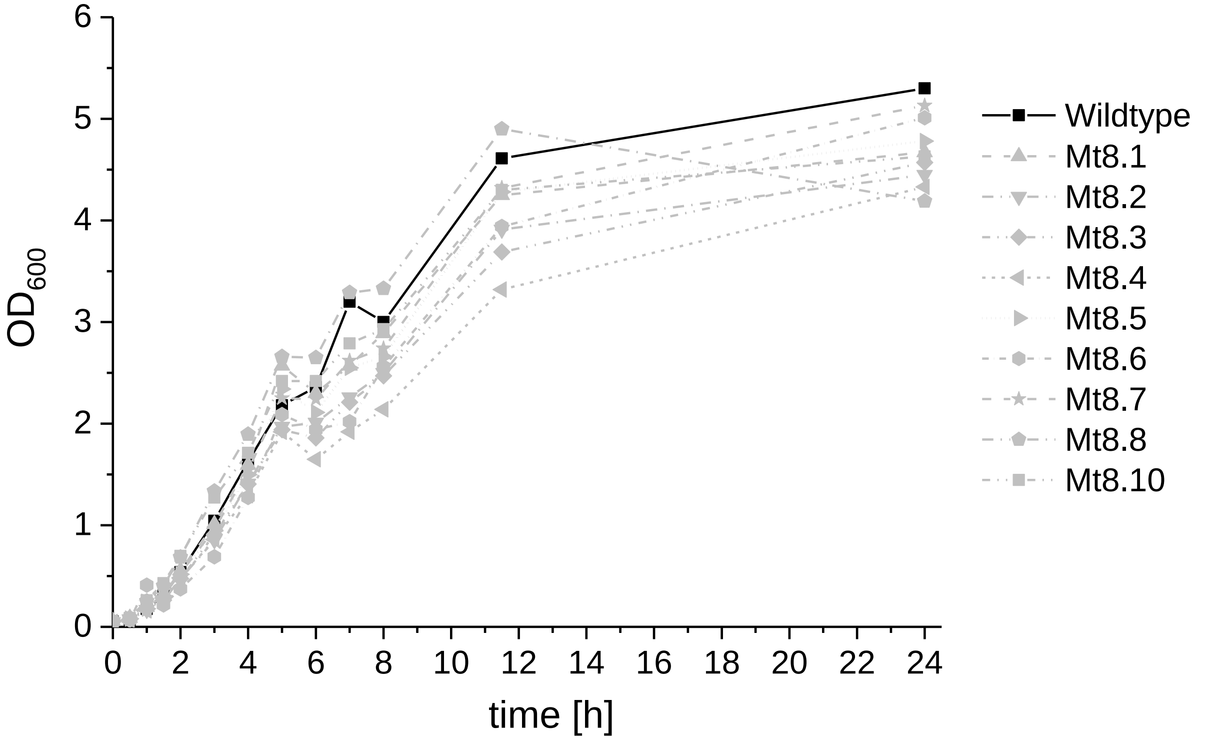

Figure S2. Growth curves of *K. pneumoniae* DSM-30104 wild type and CHD-resistant mutants (Mt8.1 – Mt8.10) over 24 h. Data represent OD_{600} values at each time point. The nine mutant strains were cultivated in presence of 8 µg/mL CHD (= 8-fold MIC).

Supplementary Tables

Table S1. MIC values of CHD, CDCHD and tetracycline (TET) for Gram-positive and Gram-negative bacteria (non-clinical strains). [a]multidrug-resistant *S. aureus*; [b]methicillin-resistant *S. aureus*; [c]vancomycin-intermediate *S. aureus*; n.d.: not determined.

Strain	MIC [µg/mL]		
	CHD	CDCHD	TET
Bacillus subtilis DSM-10	16	8	1
Enterococcus faecium DSM-20477	8	4	1
Enterococcus faecalis DSM-20478	8	4	1
Micrococcus luteus DSM-20030	32	16	4
Mycobacterium smegmatis mc^2155	4	4	n.d.
Mycobacterium bovis BCG DSM-43990	1	4	n.d.
Staphylococcus aureus			
DSM-346	4	2	0.125
DSM-11822[a]	4	2	n.d.
ATCC-29213	4	8	0.125
Newman	4	4	0.125
N315[b]	4	4	0.125
Mu50[b,c]	2	4	0.125
Staphylococcus carnosus DSM-20105	4	4	0.25
Citrobacter freundii DSM-30039	1	1	2
Enterobacter aerogenes DSM-30053	16	8	32
Escherichia coli			
DSM-1116	1	0.5	0.5
ATCC-25922	2	2	n.d.
TolC-deficient	0.5	0.25	0.5
Haemophilus influenzae DSM-11970	4	1	2
Klebsiella pneumoniae DSM-30104	1	0.5	0.5
Proteus vulgaris DSM-2140	0.25	0.25	1
Proteus mirabilis DSM-4479	0.5	1	4
Pseudomonas aeruginosa			
DSM-11128	32	16	64
DSM-24599	64	16	32
PA14	32	4	32
PA14 Δ*mexAB*	2	0.5	2
PA14 Δ*mexCD*	32	8	n.d.
PA14 Δ*mexEF*	16	8	n.d.
PA14 Δ*mexXY*	32	4	n.d.
Serratia marcescens DSM-30121	4	2	32

Table S2. Minimal inhibitory concentrations (MICs) and minimum bactericidal concentrations (MBCs) of CHD and CDCHD for *E. coli* and *K. pneumoniae* clinical isolates determined in artificial urine at different pH values. Values denote median of three independent measurements per isolate.

Isolate	pH	MIC [µg/mL]		MBC [µg/mL]	
		CHD	CDCHD	CHD	CDCHD
E. coli	5.5	1	0.5	4	8
	6.5	1	1	4	2
	7.5	1	1	8	4
	8.5	1	1	8	2
K. pneumoniae	5.5	1	1	8	2
	6.5	1	1	8	4
	7.5	2	2	16	4
	8.5	2	2	16	32

Table S3. Susceptibility of *K. pneumoniae* DSM-30104 wild type (Wt) and *K. pneumoniae* CHD-resistant mutants (Mt8.1 – Mt8.10) to various antibiotics.

Antibiotic compound	MIC [µg/mL]									
	Wt	Mt8.1	Mt8.2	Mt8.3	Mt8.4	Mt8.5	Mt8.6	Mt8.7	Mt8.8	Mt8.10
CHD	2	16	8	16	16	32	16	16	16	8
CDCHD	2	4	4	4	4	4	4	4	4	4
Tetracycline	4	64	32	64	64	64	64	64	16	32
Minocycline	4	64	64	64	64	64	> 64	64	> 64	64
Oxytetracycline	2	32	32	32	32	32	16	32	16	8
Tigecycline	0.125	1	2	2	2	1	2	2	1	4
Rifampicin	8	16	16	16	16	16	8	16	8	8
Kanamycin	2	2	2	1	1	1	0.5	2	1	0.5
Erythromycin	8	32	32	16	32	32	32	8	32	32
Polymyxin B	4	0.5	0.5	0.25	0.25	0.5	0.5	1	1	0.5
Chloramphenicol	1	32	32	32	16	8	8	16	16	8
Ciprofloxacin	< 0.03	0.125	0.025	0.125	0.125	0.125	0.125	0.125	0.125	0.125
Vancomycin	> 64	> 64	64	64	64	64	64	> 64	> 64	> 64
Ampicillin	> 64	> 64	> 64	> 64	> 64	> 64	> 64	> 64	> 64	> 64

Table S4. Mutations identified in *K. pneumoniae* DSM-30104 CHD-resistant mutants (Mt8.1 – Mt8.10) by whole genome sequencing. bp: base pair; Ins: insertion; Δ: deletion; #: number of affected bp; RE: repeat expansion. Change of codon function indicated by respective amino acids (in one letter code).

RefSeq accession	Gene product	Mutation								
		Mt8.1	Mt8.2	Mt8.3	Mt8.4	Mt8.5	Mt8.6	Mt8.7	Mt8.8	Mt8.10
WP_048253720.1	RamR: TetR/AcrR family transcriptional regulator	11bp Ins	Δ4bp	Δ4bp	Δ4bp	Δ1bp (#550); 11bp Ins	Δ1bp (#550)	11bp Ins	S137L	Δ1bp (#550)
WP_002895089.1	Phospho-glycerate mutase		2x → 3x 7bp RE	2x → 3x 7bp RE						
WP_002914333.1	ABC transporter permease (AzlC family)							G231A		

Table S5. Relative transcription levels of *ramA*, *acrA* and *acrB* genes of *K. pneumoniae* CHD-resistant mutants in comparison to *K. pneumoniae* DSM-30104 wild type (analyzed by qPCR). n.d.: not determined.

K. pneumoniae DSM-30104	*ramR* mutations	MIC [µg/mL] CHD	Relative transcription level (fold-change)		
			ramA	*acrA*	*acrB*
Wild type		2 - 4	1	1	1
KPΔr*amR*	Δr*amR*	32	17.43 ± 2.42	1.38 ± 1.17	19.75 ± 0.73
Mt8.1	11bp ins	16	18.47 ± 0.87	1.58 ± 0.74	n.d.
Mt8.2	Δ4bp	8	41.08 ± 1.05	11.78 ± 1.89	66.64 ± 0.58
Mt8.3	Δ4bp	16	14.01 ± 0.91	2.76 ± 0.73	n.d.
Mt8.4	Δ4bp	16	8.19 ± 0.55	8.40 ± 1.07	4.91 ± 0.80
Mt8.5	Δ1bp; 11bp Ins	32	23.46 ± 1.11	2.58 ± 0.71	1.41 ± 0.65
Mt8.6	Δ1bp	16	31.13 ± 1.30	4.33 ± 1.97	1.96 ± 1.13
Mt8.7	11bp Ins	16	17.78 ± 0.94	4.62 ± 2.11	1.28 ± 1.13
Mt8.8	Ser → Leu	16	3.16 ± 0.97	9.37 ± 0.71	1.47 ± 0.94
Mt8.10	Δ1bp	8	10.40 ± 1.08	3.13 ± 1.87	1.28 ± 0.85

Table S6. Activity of *K. pneumoniae* DSM-30104 wild type and CHD-resistant mutants in the presence of phenylalanine arginine β-naphthylamide dihydrochloride (PAβN). TET: tetracycline; TIG: tigecycline; CM: chloramphenicol; CIP: ciprofloxacin.

K. pneumoniae DSM-30104	PAβN	MIC [µg/ml]					
		CHD	CDCHD	TET	TIG	CM	CIP
Wildtype	-	2	1	2	0.125	1	≤ 0.03
	+	2	1	2	0.25	2	≤ 0.03
Δ*ramR*	-	16	4	8	2	8	0.06
	+	8	2	4	0.25	1	≤ 0.03
Mt8.1	-	8	2	16	1	32	0.125
	+	8	2	4	0.125	2	≤ 0.03
Mt8.2	-	16	2	32	2	32	0.25
	+	8	2	4	0.5	2	≤ 0.03
Mt8.3	-	16	2	32	2	32	0.125
	+	8	2	4	0.125	1	≤0.03
Mt8.4	-	16	2	16	2	16	0.125
	+	2	2	4	0.25	1	≤ 0.03
Mt8.5	-	16	2	16	1	8	0.125
	+	4	2	4	0.25	0.5	≤ 0.03
Mt8.6	-	16	2	16	2	8	0.125
	+	4	2	2	0.5	1	≤ 0.03
Mt8.7	-	16	2	32	2	16	0.125
	+	2	1	4	0.125	2	≤ 0.03
Mt8.8	-	8	2	16	1	16	0.125
	+	4	1	2	0.125	1	≤ 0.03
Mt8.10	-	16	2	32	4	8	0.125
	+	8	1	4	0.5	1	≤ 0.03

Table S7. MIC values of CHD-resistant mutants (selected strains C2087 - C2100) developed from *A. sulphurea* Δ*chdPKS* Δ*chdAR* (parent strain); determined in TSB medium.

Antibiotic compound	MIC [µg/mL]									
	parent	C2087	C2088	C2092	C2094	C2095	C2096	C2098	C2099	C2100
CHD	2.5	10	15	10	20	20	10	15	20	10
CDCHD	4	6	6	3	10	10	7	5	7	8

Table S8. Mutations identified in *A. sulphurea* Δ*chdPKS* Δ*chdAR* CHD-resistant mutants (strains C2087 - C2100, see Table S7) by whole genome sequencing. Change of codon function indicated by codon number and respective amino acids (in one letter code).

RefSeq accession	Gene product	Mutation								
		C2087	C2088	C2092	C2094	C2095	C2096	C2098	C2099	C2100
PFG48851.1	AfsR/SARP regulator	G317D	R675S	R645C	R645C	L469F	L469F	G671R	F261L	L469F
PFG48186.1	Sensor kinase						S187L			
PFG50225.1	Glutamate dehydrogen-ase									A1321A

Table S9. Primers for *K. pneumoniae ramR* and *A. sulphurea chdAR* gene deletions and vector constructs.

**Primer**	**Sequence (5’ → 3’ direction)**
RamRKO_fwd	CCTGGTCAGACGTGCCAAGATCGGCGGTTTGTTTAAACCTGCGTGAG GAAAAAAGTAGTGATTCCGGGGATCCGTCGACC
RamRKO_rev	CGATACGGTGAGCGCAGGGATGCAGCATCTCAGGGGTCATTTGGCG TCCGCCTCATGCAGTGTAGGCTGGAGCTGCTTC
RamRconf_fwd	GATATAACTTGATTATGAGT
RamRconf_rev	GCCCGCGAATAGTCATGGT
chdAR	TATATAGAATTCCGAGTTCGTCAAGGCGACC
chdRR	TATATATCTAGAGGACCTCCGCATCAGGC
chdRF	TATATACATATGAAGGACAATCTCGCGAGA
chdARLF	TATATAGCATGCGACGAGTCCTGGCTGTCCAC
chdARLR	TATATAACTAGTCACGCACTGGTGGATCGTC
chdARRF	TATATACATATGTGTGATCGACGAGCAGCG
chdARRR	TATATAGAATTCGACGTCCTGCTGACCGTTTC
DrrAF	TATATACATATGTCACACGCGATCCGG
DrrBR	TATATATCTAGACCGCGGACCTCAGACG

Table S10. Primers used for qPCR.

**Primer**	**Sequence (5’ → 3’ direction)**	**Target Gene**
RamA_fwd<br>RamA_rev	GGCATCTGCAACGGCTG<br>GCAGCAGCTTCCTTTCGC	*ramA*
AcrA_fwd<br>AcrA_rev	ACCAAAGTCACCTCGCCG<br>TGTTGCGGTACCAGCAGG	*acrA*
AcrB_fwd<br>AcrB_rev	GGACGGTTCCCAGGTTCG<br>TTTTCCTCACCCGGACGC	*acrB*
16S_fwd<br>16S_rev	ACGGGCGGTGTGTACAAG<br>GGCCCCCTGGACAAAGAC	16S rRNA
